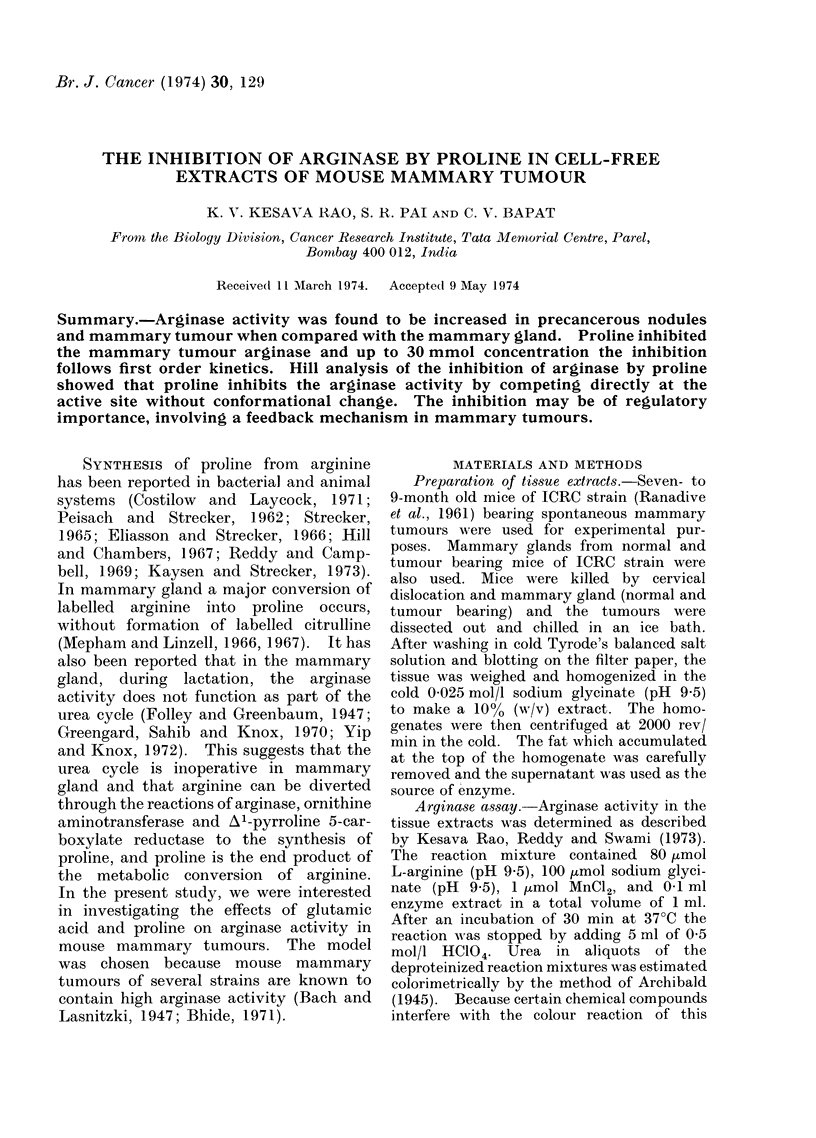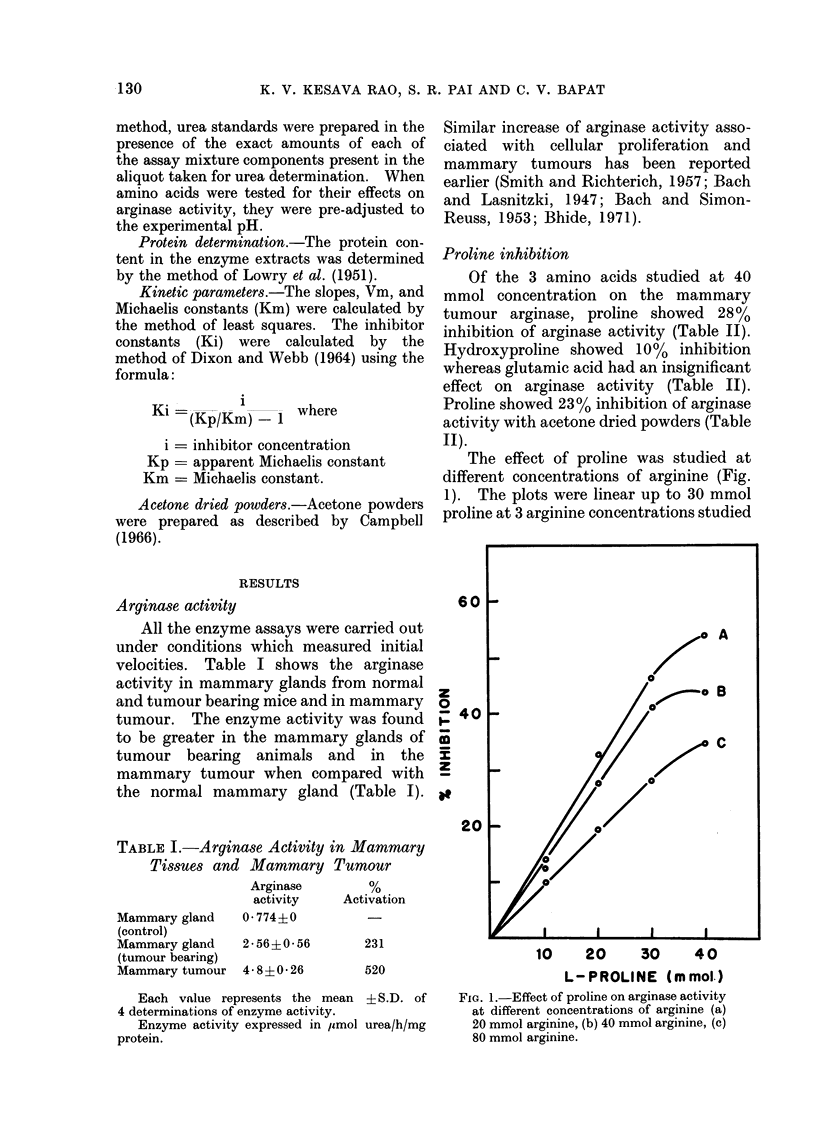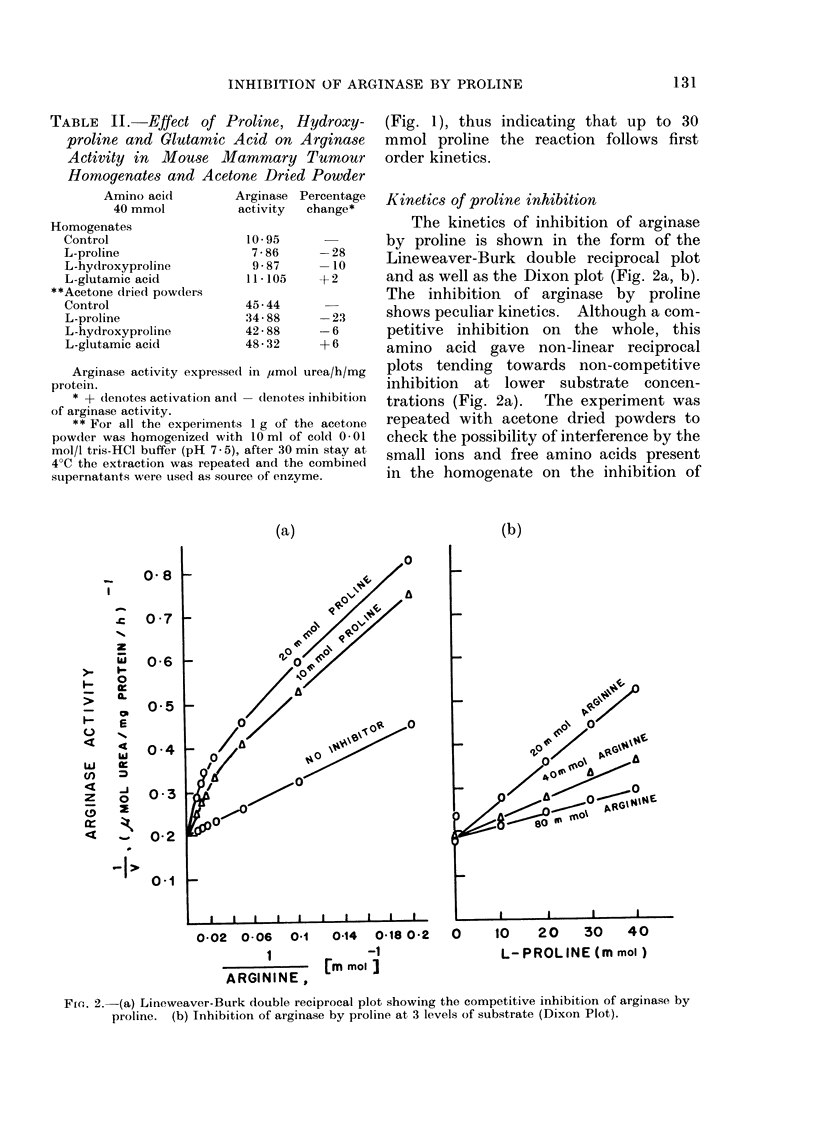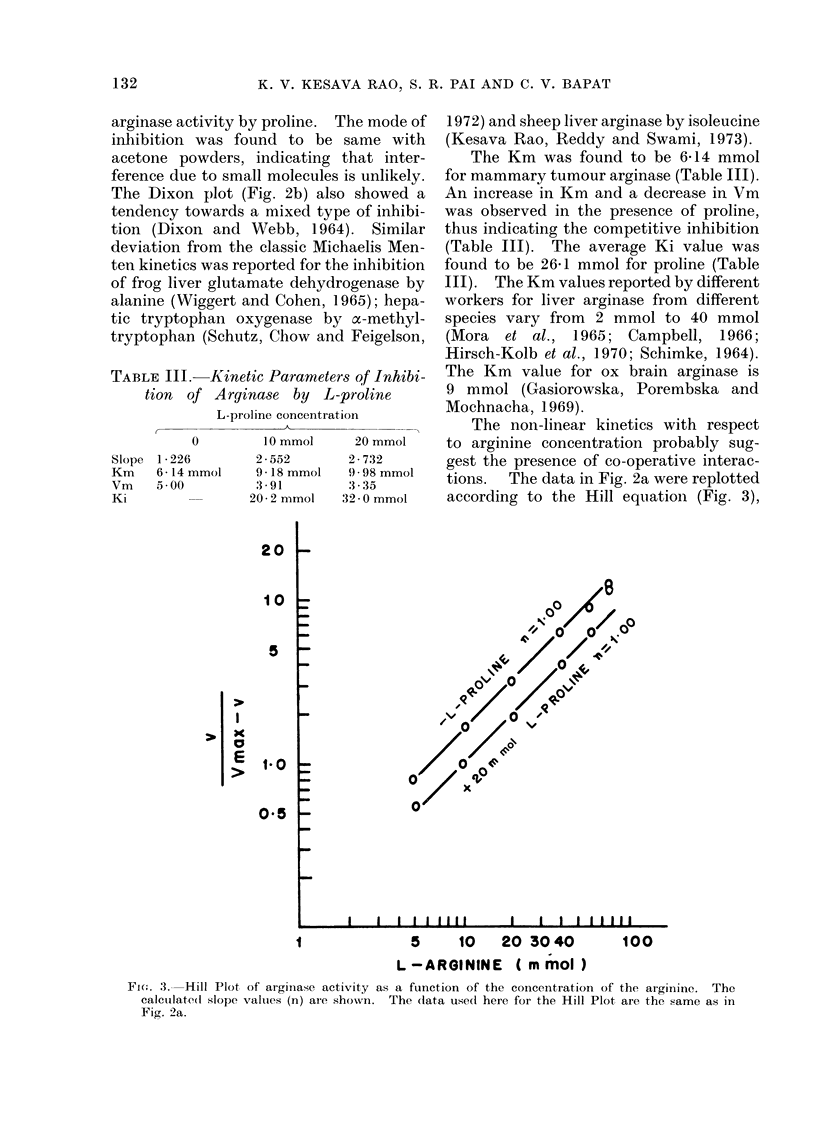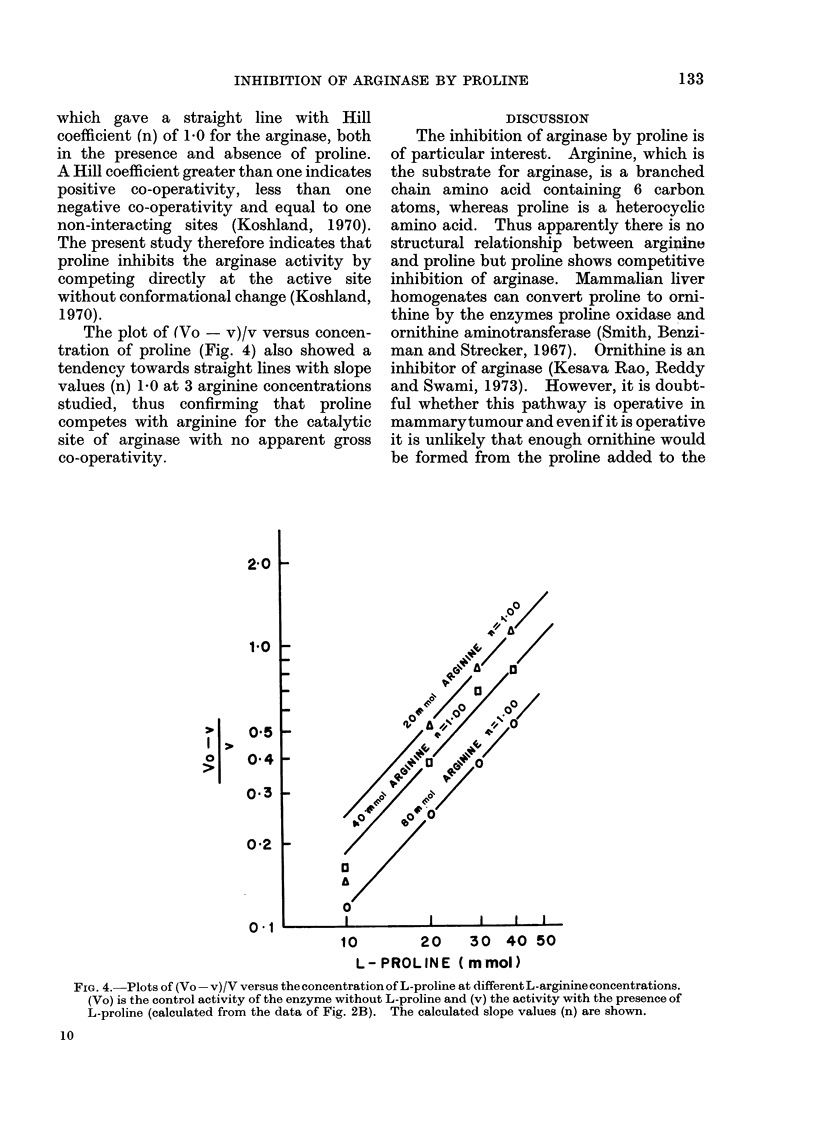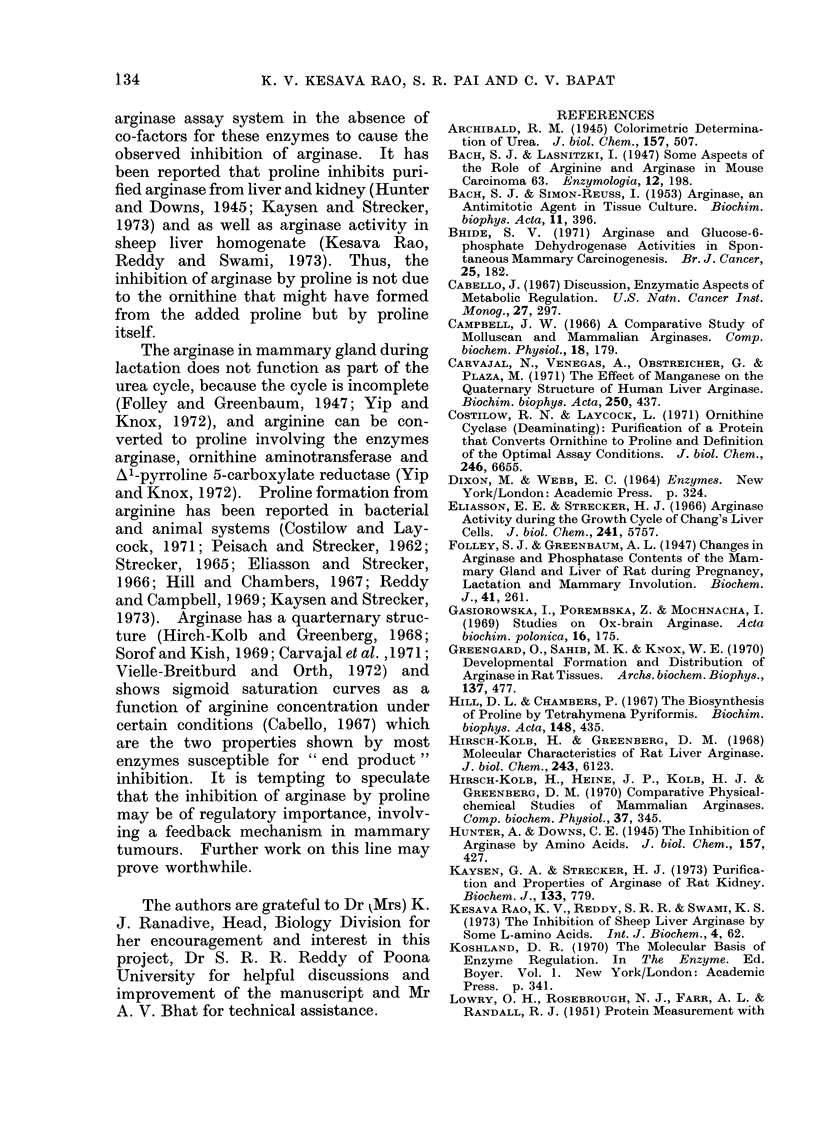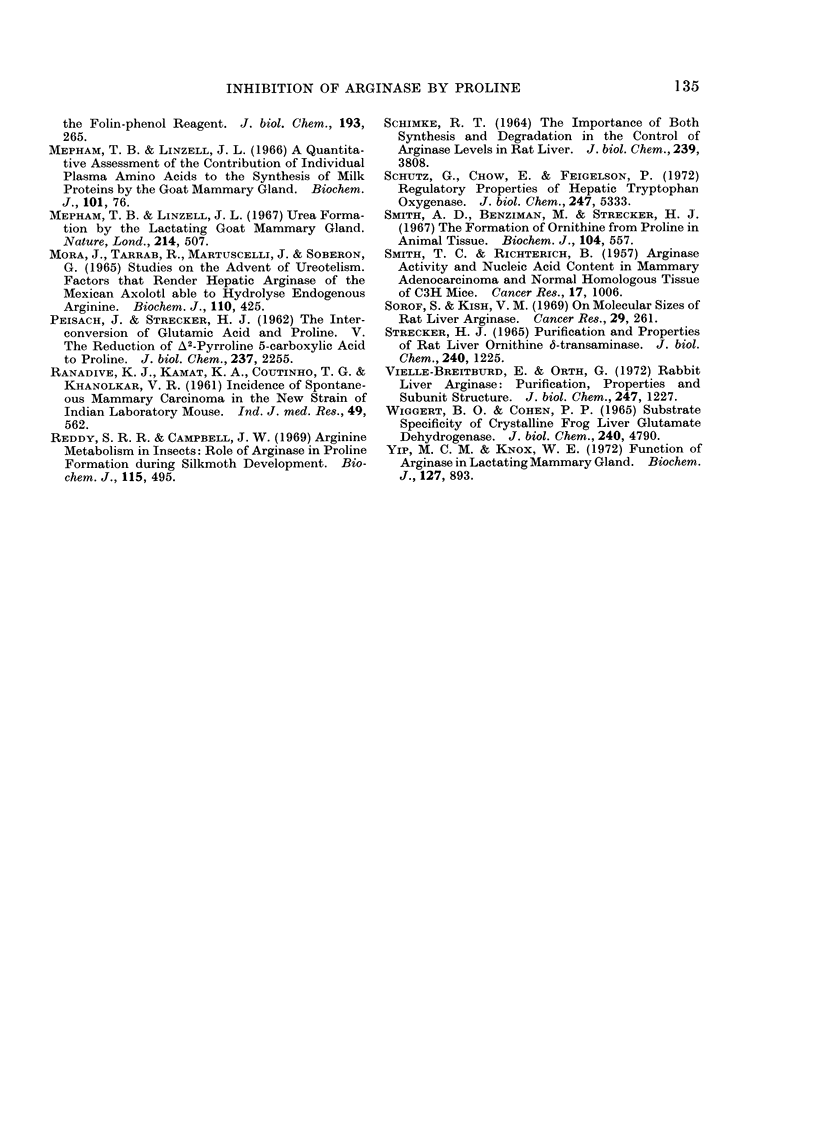# The Inhibition of Arginase by Proline in Cell-free Extracts of Mouse Mammary Tumour

**DOI:** 10.1038/bjc.1974.123

**Published:** 1974-08

**Authors:** K. V. Kesava Rao, S. R. Pai, C. V. Bapat

## Abstract

Arginase activity was found to be increased in precancerous nodules and mammary tumour when compared with the mammary gland. Proline inhibited the mammary tumour arginase and up to 30 mmo1 concentration the inhibition follows first order kinetics. Hill analysis of the inhibition of arginase by proline showed that proline inhibits the arginase activity by competing directly at the active site without conformational change. The inhibition may be of regulatory importanec, involving a feedback mechenism in mammary tumours.


*Br. J. Cancer* (1974) **30**, 129

### THE INHIBITION OF ARGINASE BY PROLINE IN CELL-FREE EXTRACTS OF MOUSE MAMMARY TUMOUR

K. V. KESAVA RAO, S. R. PAI AND C. V. BAPAT

*From the Biology Division, Cancer Research Institute, Tata Memorial Centre, Parel, Bombay* 400 012, *India*

Received 11 March 1974. Accepted 9 May 1974

**Summary.—Arginase activity was found to be increased in precancerous nodules and mammary tumour when compared with the mammary gland. Proline inhibited the mammary tumour arginase and up to 30 mmol concentration the inhibition follows first order kinetics. Hill analysis of the inhibition of arginase by proline showed that proline inhibits the arginase activity by competing directly at the active site without conformational change. The inhibition may be of regulatory importance, involving a feedback mechanism in mammary tumours.**

SYNTHESIS of proline from arginine has been reported in bacterial and animal systems (Costilow and Laycock, 1971; Peisach and Strecker, 1962; Strecker, 1965; Eliasson and Strecker, 1966; Hill and Chambers, 1967; Reddy and Campbell, 1969; Kaysen and Strecker, 1973). In mammary gland a major conversion of labelled arginine into proline occurs, without formation of labelled citrulline (Mepham and Linzell, 1966, 1967). It has also been reported that in the mammary gland, during lactation, the arginase activity does not function as part of the urea cycle (Folley and Greenbaum, 1947; Greengard, Sahib and Knox, 1970; Yip and Knox, 1972). This suggests that the urea cycle is inoperative in mammary gland and that arginine can be diverted through the reactions of arginase, ornithine aminotransferase and $\Delta^1$-pyrroline 5-carboxylate reductase to the synthesis of proline, and proline is the end product of the metabolic conversion of arginine. In the present study, we were interested in investigating the effects of glutamic acid and proline on arginase activity in mouse mammary tumours. The model was chosen because mouse mammary tumours of several strains are known to contain high arginase activity (Bach and Lasnitzki, 1947; Bhide, 1971).

### MATERIALS AND METHODS

*Preparation of tissue extracts.*—Seven- to 9-month old mice of ICRC strain (Ranadive *et al.*, 1961) bearing spontaneous mammary tumours were used for experimental purposes. Mammary glands from normal and tumour bearing mice of ICRC strain were also used. Mice were killed by cervical dislocation and mammary gland (normal and tumour bearing) and the tumours were dissected out and chilled in an ice bath. After washing in cold Tyrode's balanced salt solution and blotting on the filter paper, the tissue was weighed and homogenized in the cold 0·025 mol/l sodium glycinate (pH 9·5) to make a 10% (w/v) extract. The homogenates were then centrifuged at 2000 rev/min in the cold. The fat which accumulated at the top of the homogenate was carefully removed and the supernatant was used as the source of enzyme.

*Arginase assay.*—Arginase activity in the tissue extracts was determined as described by Kesava Rao, Reddy and Swami (1973). The reaction mixture contained 80 $\mu$mol L-arginine (pH 9·5), 100 $\mu$mol sodium glycinate (pH 9·5), 1 $\mu$mol $MnCl_2$, and 0·1 ml enzyme extract in a total volume of 1 ml. After an incubation of 30 min at 37°C the reaction was stopped by adding 5 ml of 0·5 mol/l $HClO_4$. Urea in aliquots of the deproteinized reaction mixtures was estimated colorimetrically by the method of Archibald (1945). Because certain chemical compounds interfere with the colour reaction of this method, urea standards were prepared in the presence of the exact amounts of each of the assay mixture components present in the aliquot taken for urea determination. When amino acids were tested for their effects on arginase activity, they were pre-adjusted to the experimental pH.

*Protein determination.*—The protein content in the enzyme extracts was determined by the method of Lowry *et al.* (1951).

*Kinetic parameters.*—The slopes, Vm, and Michaelis constants (Km) were calculated by the method of least squares. The inhibitor constants (Ki) were calculated by the method of Dixon and Webb (1964) using the formula:

$$Ki = \frac{i}{(Kp/Km) - 1} \quad \text{where}$$

i = inhibitor concentration
Kp = apparent Michaelis constant
Km = Michaelis constant.

*Acetone dried powders.*—Acetone powders were prepared as described by Campbell (1966).

RESULTS

### *Arginase activity*

All the enzyme assays were carried out under conditions which measured initial velocities. Table I shows the arginase activity in mammary glands from normal and tumour bearing mice and in mammary tumour. The enzyme activity was found to be greater in the mammary glands of tumour bearing animals and in the mammary tumour when compared with the normal mammary gland (Table I).

TABLE I.—*Arginase Activity in Mammary Tissues and Mammary Tumour*

| | Arginase activity | % Activation |
|---|---|---|
| Mammary gland (control) | 0·774±0 | — |
| Mammary gland (tumour bearing) | 2·56±0·56 | 231 |
| Mammary tumour | 4·8±0·26 | 520 |

Each value represents the mean ±S.D. of 4 determinations of enzyme activity.

Enzyme activity expressed in μmol urea/h/mg protein.

Similar increase of arginase activity associated with cellular proliferation and mammary tumours has been reported earlier (Smith and Richterich, 1957; Bach and Lasnitzki, 1947; Bach and Simon-Reuss, 1953; Bhide, 1971).

### *Proline inhibition*

Of the 3 amino acids studied at 40 mmol concentration on the mammary tumour arginase, proline showed 28% inhibition of arginase activity (Table II). Hydroxyproline showed 10% inhibition whereas glutamic acid had an insignificant effect on arginase activity (Table II). Proline showed 23% inhibition of arginase activity with acetone dried powders (Table II).

The effect of proline was studied at different concentrations of arginine (Fig. 1). The plots were linear up to 30 mmol proline at 3 arginine concentrations studied

FIG. 1.—Effect of proline on arginase activity at different concentrations of arginine (a) 20 mmol arginine, (b) 40 mmol arginine, (c) 80 mmol arginine.

TABLE II.—*Effect of Proline, Hydroxyproline and Glutamic Acid on Arginase Activity in Mouse Mammary Tumour Homogenates and Acetone Dried Powder*

| Amino acid 40 mmol | Arginase activity | Percentage change* |
|---|---|---|
| Homogenates | | |
| Control | 10·95 | — |
| L-proline | 7·86 | −28 |
| L-hydroxyproline | 9·87 | −10 |
| L-glutamic acid | 11·105 | +2 |
| **Acetone dried powders | | |
| Control | 45·44 | — |
| L-proline | 34·88 | −23 |
| L-hydroxyproline | 42·88 | −6 |
| L-glutamic acid | 48·32 | +6 |

Arginase activity expressed in μmol urea/h/mg protein.

* + denotes activation and − denotes inhibition of arginase activity.

** For all the experiments 1 g of the acetone powder was homogenized with 10 ml of cold 0·01 mol/l tris-HCl buffer (pH 7·5), after 30 min stay at 4°C the extraction was repeated and the combined supernatants were used as source of enzyme.

(Fig. 1), thus indicating that up to 30 mmol proline the reaction follows first order kinetics.

*Kinetics of proline inhibition*

The kinetics of inhibition of arginase by proline is shown in the form of the Lineweaver-Burk double reciprocal plot and as well as the Dixon plot (Fig. 2a, b). The inhibition of arginase by proline shows peculiar kinetics. Although a competitive inhibition on the whole, this amino acid gave non-linear reciprocal plots tending towards non-competitive inhibition at lower substrate concentrations (Fig. 2a). The experiment was repeated with acetone dried powders to check the possibility of interference by the small ions and free amino acids present in the homogenate on the inhibition of

FIG. 2.—(a) Lineweaver-Burk double reciprocal plot showing the competitive inhibition of arginase by proline. (b) Inhibition of arginase by proline at 3 levels of substrate (Dixon Plot).

arginase activity by proline. The mode of inhibition was found to be same with acetone powders, indicating that interference due to small molecules is unlikely. The Dixon plot (Fig. 2b) also showed a tendency towards a mixed type of inhibition (Dixon and Webb, 1964). Similar deviation from the classic Michaelis Menten kinetics was reported for the inhibition of frog liver glutamate dehydrogenase by alanine (Wiggert and Cohen, 1965); hepatic tryptophan oxygenase by $\alpha$-methyltryptophan (Schutz, Chow and Feigelson, 1972) and sheep liver arginase by isoleucine (Kesava Rao, Reddy and Swami, 1973).

The Km was found to be 6·14 mmol for mammary tumour arginase (Table III). An increase in Km and a decrease in Vm was observed in the presence of proline, thus indicating the competitive inhibition (Table III). The average Ki value was found to be 26·1 mmol for proline (Table III). The Km values reported by different workers for liver arginase from different species vary from 2 mmol to 40 mmol (Mora *et al.*, 1965; Campbell, 1966; Hirsch-Kolb *et al.*, 1970; Schimke, 1964). The Km value for ox brain arginase is 9 mmol (Gasiorowska, Porembska and Mochnacha, 1969).

The non-linear kinetics with respect to arginine concentration probably suggest the presence of co-operative interactions. The data in Fig. 2a were replotted according to the Hill equation (Fig. 3),

TABLE III.—*Kinetic Parameters of Inhibition of Arginase by L-proline*

| | L-proline concentration | | |
|---|---|---|---|
| | 0 | 10 mmol | 20 mmol |
| Slope | 1·226 | 2·552 | 2·732 |
| Km | 6·14 mmol | 9·18 mmol | 9·98 mmol |
| Vm | 5·00 | 3·91 | 3·35 |
| Ki | — | 20·2 mmol | 32·0 mmol |

FIG. 3.—Hill Plot of arginase activity as a function of the concentration of the arginine. The calculated slope values (n) are shown. The data used here for the Hill Plot are the same as in Fig. 2a.

which gave a straight line with Hill coefficient (n) of 1·0 for the arginase, both in the presence and absence of proline. A Hill coefficient greater than one indicates positive co-operativity, less than one negative co-operativity and equal to one non-interacting sites (Koshland, 1970). The present study therefore indicates that proline inhibits the arginase activity by competing directly at the active site without conformational change (Koshland, 1970).

The plot of (Vo — v)/v versus concentration of proline (Fig. 4) also showed a tendency towards straight lines with slope values (n) 1·0 at 3 arginine concentrations studied, thus confirming that proline competes with arginine for the catalytic site of arginase with no apparent gross co-operativity.

DISCUSSION

The inhibition of arginase by proline is of particular interest. Arginine, which is the substrate for arginase, is a branched chain amino acid containing 6 carbon atoms, whereas proline is a heterocyclic amino acid. Thus apparently there is no structural relationship between arginine and proline but proline shows competitive inhibition of arginase. Mammalian liver homogenates can convert proline to ornithine by the enzymes proline oxidase and ornithine aminotransferase (Smith, Benziman and Strecker, 1967). Ornithine is an inhibitor of arginase (Kesava Rao, Reddy and Swami, 1973). However, it is doubtful whether this pathway is operative in mammary tumour and even if it is operative it is unlikely that enough ornithine would be formed from the proline added to the

FIG. 4.—Plots of (Vo — v)/V versus the concentration of L-proline at different L-arginine concentrations. (Vo) is the control activity of the enzyme without L-proline and (v) the activity with the presence of L-proline (calculated from the data of Fig. 2B). The calculated slope values (n) are shown.

arginase assay system in the absence of co-factors for these enzymes to cause the observed inhibition of arginase. It has been reported that proline inhibits purified arginase from liver and kidney (Hunter and Downs, 1945; Kaysen and Strecker, 1973) and as well as arginase activity in sheep liver homogenate (Kesava Rao, Reddy and Swami, 1973). Thus, the inhibition of arginase by proline is not due to the ornithine that might have formed from the added proline but by proline itself.

The arginase in mammary gland during lactation does not function as part of the urea cycle, because the cycle is incomplete (Folley and Greenbaum, 1947; Yip and Knox, 1972), and arginine can be converted to proline involving the enzymes arginase, ornithine aminotransferase and $\Delta^1$-pyrroline 5-carboxylate reductase (Yip and Knox, 1972). Proline formation from arginine has been reported in bacterial and animal systems (Costilow and Laycock, 1971; Peisach and Strecker, 1962; Strecker, 1965; Eliasson and Strecker, 1966; Hill and Chambers, 1967; Reddy and Campbell, 1969; Kaysen and Strecker, 1973). Arginase has a quarternary structure (Hirch-Kolb and Greenberg, 1968; Sorof and Kish, 1969; Carvajal *et al.* ,1971; Vielle-Breitburd and Orth, 1972) and shows sigmoid saturation curves as a function of arginine concentration under certain conditions (Cabello, 1967) which are the two properties shown by most enzymes susceptible for "end product" inhibition. It is tempting to speculate that the inhibition of arginase by proline may be of regulatory importance, involving a feedback mechanism in mammary tumours. Further work on this line may prove worthwhile.

The authors are grateful to Dr (Mrs) K. J. Ranadive, Head, Biology Division for her encouragement and interest in this project, Dr S. R. R. Reddy of Poona University for helpful discussions and improvement of the manuscript and Mr A. V. Bhat for technical assistance.

### REFERENCES

ARCHIBALD, R. M. (1945) Colorimetric Determination of Urea. *J. biol. Chem.*, **157**, 507.

BACH, S. J. & LASNITZKI, I. (1947) Some Aspects of the Role of Arginine and Arginase in Mouse Carcinoma 63. *Enzymologia*, **12**, 198.

BACH, S. J. & SIMON-REUSS, I. (1953) Arginase, an Antimitotic Agent in Tissue Culture. *Biochim. biophys. Acta*, **11**, 396.

BHIDE, S. V. (1971) Arginase and Glucose-6-phosphate Dehydrogenase Activities in Spontaneous Mammary Carcinogenesis. *Br. J. Cancer*, **25**, 182.

CABELLO, J. (1967) Discussion, Enzymatic Aspects of Metabolic Regulation. *U.S. Natn. Cancer Inst. Monog.*, **27**, 297.

CAMPBELL, J. W. (1966) A Comparative Study of Molluscan and Mammalian Arginases. *Comp. biochem. Physiol.*, **18**, 179.

CARVAJAL, N., VENEGAS, A., OBSTREICHER, G. & PLAZA, M. (1971) The Effect of Manganese on the Quaternary Structure of Human Liver Arginase. *Biochim. biophys. Acta*, **250**, 437.

COSTILOW, R. N. & LAYCOCK, L. (1971) Ornithine Cyclase (Deaminating): Purification of a Protein that Converts Ornithine to Proline and Definition of the Optimal Assay Conditions. *J. biol. Chem.*, **246**, 6655.

DIXON, M. & WEBB, E. C. (1964) *Enzymes.* New York/London: Academic Press. p. 324.

ELIASSON, E. E. & STRECKER, H. J. (1966) Arginase Activity during the Growth Cycle of Chang's Liver Cells. *J. biol. Chem.*, **241**, 5757.

FOLLEY, S. J. & GREENBAUM, A. L. (1947) Changes in Arginase and Phosphatase Contents of the Mammary Gland and Liver of Rat during Pregnancy, Lactation and Mammary Involution. *Biochem. J.*, **41**, 261.

GASIOROWSKA, I., POREMBSKA, Z. & MOCHNACHA, I. (1969) Studies on Ox-brain Arginase. *Acta biochim. polonica*, **16**, 175.

GREENGARD, O., SAHIB, M. K. & KNOX, W. E. (1970) Developmental Formation and Distribution of Arginase in Rat Tissues. *Archs. biochem. Biophys.*, **137**, 477.

HILL, D. L. & CHAMBERS, P. (1967) The Biosynthesis of Proline by Tetrahymena Pyriformis. *Biochim. biophys. Acta*, **148**, 435.

HIRSCH-KOLB, H. & GREENBERG, D. M. (1968) Molecular Characteristics of Rat Liver Arginase. *J. biol. Chem.*, **243**, 6123.

HIRSCH-KOLB, H., HEINE, J. P., KOLB, H. J. & GREENBERG, D. M. (1970) Comparative Physical-chemical Studies of Mammalian Arginases. *Comp. biochem. Physiol.*, **37**, 345.

HUNTER, A. & DOWNS, C. E. (1945) The Inhibition of Arginase by Amino Acids. *J. biol. Chem.*, **157**, 427.

KAYSEN, G. A. & STRECKER, H. J. (1973) Purification and Properties of Arginase of Rat Kidney. *Biochem. J.*, **133**, 779.

KESAVA RAO, K. V., REDDY, S. R. R. & SWAMI, K. S. (1973) The Inhibition of Sheep Liver Arginase by Some L-amino Acids. *Int. J. Biochem.*, **4**, 62.

KOSHLAND, D. R. (1970) The Molecular Basis of Enzyme Regulation. In *The Enzyme.* Ed. Boyer. Vol. 1. New York/London: Academic Press. p. 341.

LOWRY, O. H., ROSEBROUGH, N. J., FARR, A. L. & RANDALL, R. J. (1951) Protein Measurement with the Folin-phenol Reagent. *J. biol. Chem.*, **193**, 265.

Mepham, T. B. & Linzell, J. L. (1966) A Quantitative Assessment of the Contribution of Individual Plasma Amino Acids to the Synthesis of Milk Proteins by the Goat Mammary Gland. *Biochem. J.*, **101**, 76.

Mepham, T. B. & Linzell, J. L. (1967) Urea Formation by the Lactating Goat Mammary Gland. *Nature, Lond.*, **214**, 507.

Mora, J., Tarrab, R., Martuscelli, J. & Soberon, G. (1965) Studies on the Advent of Ureotelism. Factors that Render Hepatic Arginase of the Mexican Axolotl able to Hydrolyse Endogenous Arginine. *Biochem. J.*, **110**, 425.

Peisach, J. & Strecker, H. J. (1962) The Interconversion of Glutamic Acid and Proline. V. The Reduction of $\Delta^2$-Pyrroline 5-carboxylic Acid to Proline. *J. biol. Chem.*, **237**, 2255.

Ranadive, K. J., Kamat, K. A., Coutinho, T. G. & Khanolkar, V. R. (1961) Incidence of Spontaneous Mammary Carcinoma in the New Strain of Indian Laboratory Mouse. *Ind. J. med. Res.*, **49**, 562.

Reddy, S. R. R. & Campbell, J. W. (1969) Arginine Metabolism in Insects: Role of Arginase in Proline Formation during Silkmoth Development. *Biochem. J.*, **115**, 495.

Schimke, R. T. (1964) The Importance of Both Synthesis and Degradation in the Control of Arginase Levels in Rat Liver. *J. biol. Chem.*, **239**, 3808.

Schutz, G., Chow, E. & Feigelson, P. (1972) Regulatory Properties of Hepatic Tryptophan Oxygenase. *J. biol. Chem.*, **247**, 5333.

Smith, A. D., Benziman, M. & Strecker, H. J. (1967) The Formation of Ornithine from Proline in Animal Tissue. *Biochem. J.*, **104**, 557.

Smith, T. C. & Richterich, B. (1957) Arginase Activity and Nucleic Acid Content in Mammary Adenocarcinoma and Normal Homologous Tissue of C3H Mice. *Cancer Res.*, **17**, 1006.

Sorof, S. & Kish, V. M. (1969) On Molecular Sizes of Rat Liver Arginase. *Cancer Res.*, **29**, 261.

Strecker, H. J. (1965) Purification and Properties of Rat Liver Ornithine δ-transaminase. *J. biol. Chem.*, **240**, 1225.

Vielle-Breitburd, E. & Orth, G. (1972) Rabbit Liver Arginase: Purification, Properties and Subunit Structure. *J. biol. Chem.*, **247**, 1227.

Wiggert, B. O. & Cohen, P. P. (1965) Substrate Specificity of Crystalline Frog Liver Glutamate Dehydrogenase. *J. biol. Chem.*, **240**, 4790.

Yip, M. C. M. & Knox, W. E. (1972) Function of Arginase in Lactating Mammary Gland. *Biochem. J.*, **127**, 893.